# 

**Published:** 1861-01

**Authors:** 


New Series, Vol. 4. No. 1. Whole Series, Vol. 18, No. 1.

### THE CHICAGO

### MEDICAL JOURNAL.

DANIEL BRAINARD, M. D.,

J. ADAMS ALLEN, M. D.,

Editors and Proprietors.

JANUARY, 1861.

CHICAGO:
WM. PIGOTT, BOOK AND JOB PRINTER, 126 CLARK STREET.

1861.

